# Components and methodology of evidence briefs for policy: the need for evaluation tools

**DOI:** 10.1186/s12961-026-01451-y

**Published:** 2026-02-26

**Authors:** Xuping Song, Ruixin Li, Qiyin Luo, Ludovic Reveiz, Marge Reinap, Evelina Chapman, Laurenz Mahlanza-Langer, Laura Boeira, Xue Li, Xiuxia Li, Yaolong Chen, Kehu Yang, John Lavis, Tanja Kuchenmüller

**Affiliations:** 1 School of Public Health, Lanzhou University, Lanzhou, 730000 China; 2 The Centre of Evidence-based Social Science, School of Public Health, Lanzhou University, Lanzhou, China; 3 Evidence-Based Medicine Center, School of Basic Medicine, Lanzhou University, Lanzhou, China; 4 Key Laboratory of Evidence Based Medicine and Knowledge Translation of Gansu Province, Lanzhou, China; 5 WHO Collaborating Centre for Guideline Implementation and Knowledge Translation, Lanzhou, China; 6 Science and Knowledge Unit, Evidence and Intelligence for Action in Health Department, Pan American Health Organization, Washington, DC USA; 7 Division of Country Health Policies and System, World Health Organization Regional Office for Europe, Copenhagen, Denmark; 8 Faculty of Medicine of the Universidad de la Frontera, Temuco, Chile; 9 Pan American Health Organization, Columbia, Washington, DC USA; 10 Pan-African Collective for Evidence, Johannesburg, South Africa; 11 Instituto Veredas, Belo Horizonte, Brazil; 12 National Health Development Research Center, Beijing, China; 13 Health Technology Assessment Center/Evidence-Based Social Science Research Center, School of Public Health, Lanzhou University, Lanzhou, China; 14 McMaster Health Forum, McMaster University, 1280 Main Street West MML-417, Hamilton, Canada; 15 Research and Ethics Ecosystem Strengthening Science for Health, Science Division, World Health Organization, Geneva, Switzerland

**Keywords:** Evidence briefs for policy, Policy brief, Quality of methodology

## Abstract

**Background:**

Evidence briefs for policy (EBPs) are effective tools for delivering research evidence to policymakers and other stakeholders by highlighting high-priority issues, outlining options and considering implementation strategies. However, policymakers’ demands for evidence and policy-relevant information across different fields have led to variability in the terminology used to describe EBPs, and the methodological quality of these EBPs remains unclear. This study aims to (1) identify organizations whose definitions of EBPs contain the three key components of problem, options and implementation considerations, (2) assess the methodological quality of EBPs that incorporate these three key components and (3) identify existing evaluation/assessment tools of EBPs.

**Methods:**

A two-stage documentary analysis approach was used. First, we identified documents that were produced by organizations/institutions to inform policymakers and that contained the three key components (Problem, Options and Implementation considerations). Second, the methodological quality of the documents was assessed from the perspectives of the evidence supply side (that is, evidence synthesis) and the evidence demand side (that is, mapping of and engagement between both policymakers and stakeholders).

**Results:**

In 22 organizations, the term policy brief was the most commonly used, accounting for 50% of organizations, while other terms varied. Issue briefs were used by three organizations (13.6%) and evidence briefs were used by two organizations (9.1%). In total, 50 individual documents from nine different organizations were included to evaluate components and methodology. (1) From the supply-side perspective: 17 (34%) documents described the search resources, 10 (20%) documents described evidence certainty and 15 (30%) assessed the methodological quality of the research evidence. (2) From the demand-side perspective: 30 (60%) documents were developed in response to demand-side needs, while 27 (54%) included both stakeholder mapping and engagement.

**Conclusions:**

Methodological shortcomings were identified in the EBPs from both the supply-side and demand-side perspectives, highlighting the need to validate and better implement existing tools and to complement existing guidelines.

**Supplementary Information:**

The online version contains supplementary material available at 10.1186/s12961-026-01451-y.

## Introduction

Evidence briefs for policy (EBPs), also known as policy briefs or evidence briefs [1, 2], are information packaging and synthesis tools intended to provide access to or package health information in a user-friendly manner, in response to policy-makers’ needs [3]. The term was agreed upon by the steering committee of the Evidence-Informed Policy Network (EVIPNet), a global initiative led by the World Health Organization (WHO) to promote the systematic use of health research evidence in policy-making. Writing and using EBPs was formally included in the network’s strategic plan [4]. According to the purpose of their development, EBPs can be categorized into several types, including issue briefs, policy landscape briefs, modelling briefs and policy analysis briefs [5]. An EBP and dialogue summary can be used by a national government to inform a decision, making the decision higher quality and more aligned with the needs of advisory opinion and decision-making [6]. An increasing number of countries and organizations have adopted EBPs as a way to inform health policy decisions [7–9].

Adam et al. have pointed out that policy briefs are known by a variety of names. There are a few consistent names used to label and describe evidence briefs or policy briefs and many variations in the features and characteristics of EBPs [1]. Several documents have been published to guide EBP development [5, 10, 11]. For example, WHO issued a template to help write an effective EBP used in the WHO Mediterranean Region [12]. In addition, a standard reporting guideline for evidence briefs for policy (STEP) has been developed by Yu et al. [13]. Transparent reporting of the methodology used to develop EBPs is recommended by some guidance documents [14]. However, a study by Zhang et al. analysing 129 health-related EBPs found that such transparency is uncommon: only 46% described their development methodology, merely 9% reported the evidence synthesis method and 18% reported using database searches [2]. While clinical management decisions appear to follow a process for identifying, assessing and considering evidence in decision-making to a greater extent, this process is more limited for population-level interventions [15]. Quality, compelling evidence is crucial in facilitating decision-making by policymakers [16]. This is critical as we know that, for evidence to be used, policymakers need to trust the evidence and know how much confidence they can place in decision-making based on the methodological quality of EBPs. In summary, the above two issues (the confusion of terminology and that the methodology is not transparently documented) hinder policymakers from identifying and using high-quality EBPs.

Lack of consistency in the terminology used to label or describe types of documents with similar content makes them more challenging to locate and identify. To investigate this issue, we collected definitions from three different publications: SUPPORT Tools [17], SURE Guides [18] and WHO Evidence Briefs for Policy Using the Integrated Knowledge Translation Approach [14]. Following these definitions, EBPs that included the three key elements of problem (what the problem is and its context), options (present policy options rather than make direct recommendations) and implementation considerations (identify if there are potential barriers and facilitators to the successful implementation of an option or element) were selected for this study. Three core components (problem, options, implementation considerations) had to be present as labelled. Documents that did not contain all three components were excluded. Overall, this study aimed to: (1) identify organizations that use particular terms to define EBPs containing the three key components of problem, options and implementation considerations, (2) assess the methodological quality of EBPs that incorporate these three key components and (3) identify existing evaluation/assessment tools of EBPs.

## Methods

We used a two-stage approach to identify EBPs for this study, owing to EBPs being grey literature and the specificity of their search [1]. First, organizations and institutions involved in the development of EBPs were identified, followed by an analysis of documents prepared primarily to provide policymakers with information, including both research evidence and other types of policy-relevant content (see Additional file 1).

### Literature search

Google and Google Scholar were used as the search engines in this study, the top 200 records of Google and Google Scholar sort by relevance be searched, with search terms: “brief for policy”, “policy brief”, “EBP”, “evidence brief”, “issue brief”, “citizen brief”, “research brief”, “evidence-informed policy brief” and “evidence-based policy brief”. The search was conducted in English, over the period of 1 January 2019 to 30 June 2024. Then, expert consultations were conducted to verify that the organizations/institutions were valid for inclusion. A purposively selected panel of six experts was convened to serve as an Advisory Board. The panel comprised specialists in knowledge translation and science policy from diverse geographical regions (Africa, the Americas, Europe and the Western Pacific). Specifically, the advisory board with complementary expertise across: (1) methodology of knowledge translation and evidence informed policy-making, (2) standards and guidelines development, (3) EBP development and (4) impact evaluation. The advisory board reviewed and provided expert advice during the organization/institution’s identification and EBPs analysis. All documents included in this study were downloaded from the website of the organization/institution, and the full texts of the included studies were collated and coded.

### Criteria

According to the mainstream definition, EBPs are prepared by synthesizing and contextualizing the best available evidence about a problem, presenting viable solutions to address it and addressing key implementation considerations, with the involvement of content experts, policymakers and stakeholders. The inclusion criteria for documents in this study were as follows: (1) EBPs must have been prepared with the primary intention of providing information (both research evidence and other types of policy-relevant information) to policymakers and broadly aim to support policy-making, (2) must contain the three key components of problem, options and implementation considerations and (3) must have been developed or updated in the last 5 years.

The initial search identified 22 organizations that produce documents falling under the broad category of EBP. Furthermore, to ensure a focused analysis of organizations relevant to our research question, we prioritized organizations that had produced at least five documents within our study’s timeframe (2019–2024). The organizations excluded, along with the reasons for exclusion, are listed in Table 2. This step ensured a sufficient volume of material for an analysis for each organization. A total of 10 organizations were included in the in-depth study. A convenience sampling strategy was employed. For each of the 10 included organizations, we systematically identified all publicly accessible documents that met our EBP inclusion criteria on their official websites. To ensure consistency in document selection across organizations, we implemented a two-step procedure: first, documents were sorted by relevance using the organizations’ website search functions or categorization systems; second, from this relevance-ordered list, we selected up to five documents published in different years where possible, aiming to achieve broad temporal representation. This approach resulted in the final analytical sample of 50 documents.

### Screening and data extraction

After the search process, the literature screening was completed according to whether the text of the EBPs met the three criteria. The extraction table was modified and developed with reference to the extraction entries of Adam et al. and Zhang et al. [1, 2] and modified through two rounds of pre-testing. Then, a pre-developed table in Microsoft Excel 2016 was used to perform the data extraction. Extracted information included but was not limited to the following: organizations, terms, problems, options, implementation considerations, living document, development methodology, search resource, evidence certainty, quality of research evidence, living EBPs, key informant interviews and demand-side requests. Two researchers performed the data extraction independently, and a third researcher ruled on all disagreements between them.

### Data analysis

To facilitate the analysis of the retrieved documents and to develop a deeper understanding of their characteristics and methodologies, the extracted data were integrated and statistically analysed. Descriptive statistics were used to report and summarize the basic information of the included studies. Content analysis of the sampled documents began during the initial data extraction phase and followed an inductive thematic approach through multiple iterations to categorize the documents by content type, characteristics, labelling terminology and methodology. The thematic approach is a qualitative research method that involves identifying, analysing and interpreting themes within textual data to make sense of the information [19]. The data were summarized as numbers and percentages. For the qualitative analysis of elements, a “√” was used to indicate the presence of an element and an “×” to indicate its absence.

To assess the methodological and reporting characteristics of the included evidence briefs for policy (EBPs), we developed a set of ten core criteria on the basis of EBP development manuals and tried to analyse them from two different perspectives: (1) supply-side, which refers to the generation, synthesis and packaging of evidence into accessible formats for users. Our analysis of supply-side factors focuses on the methodological aspects of EBPs, such as how evidence is searched and synthesized and the evidence certainty. (2) Demand-side: this pertains to the access, interpretation and application of evidence by policymakers and other stakeholders, including the problem (problem-driven, EBP was developed at the request of the demand side) and stakeholder engagement. These criteria were designed to evaluate key domains, including the document type (main topic and living EBPs), methodological quality (for example, description of development methodology, search resources, assessment of evidence certainty and quality) and co-production (see Table 1 for full definitions) [20, 21].

**Table 1:** Extraction items and description

Extraction items	Description
Living EBPs	The EBP is continually updated as a living document [19]
Implementation considerations	The EBP identifies implementation considerations at various levels
Development methodology	The EBP provides a methodological description of its preparation in a suitable and accessible section of the document
Primary research	The development of the EBP was based on primary research or the EBP was conducted as primary research in its own right
Search resource	The EBP describes search resources for the evidence synthesis process
Evidence certainty	The EBP describes the certainty of the evidence for the evidence synthesis process
Quality of research evidence	The EBP assesses the methodological quality of the studies included in its development process
Problem	The EBP was developed at the request of the demand side (that is, government, society, organizations and so on)
Stakeholder mapping	The EBP includes stakeholder mapping. Stakeholder mapping is the process of systematically identifying and analysing key individuals or groups who have an interest in or may be affected by a policy or project, to engage them appropriately. [20]
Stakeholder engagement	The stakeholders engaged in development of the EBP through a steering group, a technical group and so on

## Results

### Terms regarding EBPs

Five documents prepared by each organization/institution were sampled, and the literature screening and data extraction were completed after the search and sample process. Within the sample of 22 organizations, eight distinct document labels were identified. Among these, policy brief was the most common term, used by 11 (50%) organizations/institutions. Other labels, issue brief (13.6%), evidence brief (9.1%) and briefing (9.1%), were less frequent. The terms evidence briefs for policy, briefs, policy briefcase and evidence-based policy briefs were each used by a single organization (each 4.5%). In total, three (13.6%) organizations/institutions used the issue briefs, two (9.1%) organizations/institutions used the evidence briefs and two (9.1%) organizations/institutions used the briefing; the use of evidence briefs for policy, briefs, policy briefcase and evidence-based policy briefs were all one (4.5%). The details of terms utilized in major organizations/institutions are shown in Table 2.

**Table 2:** EBPs with three key components

Terms	Organizations/institutions	Problem	Options	Implementation considerations	Reason for exclusion
Included organizations					
Evidence briefs for policy	Evidence-Informed Policy Network, EVIPNet	√	√	√	/
Policy briefs	The American University of Beirut, Knowledge to Policy, K2P	√	√	√	/
	World Health Organization, WHO	√	√	√	/
	European Observatory on Health Systems and Policies, OBS—Observatory	√	√	√	/
	World Bank	√	√	√	/
	Think7	√	√	√	/
Evidence briefs	McMaster Health Forum, MHF	√	√	√	/
	Department of Veterans Affairs, VA	√	√	√	/
Briefs	International Initiative for Impact Evaluation, 3ie	√	√	√	/
Briefing	The Youth Endowment Fund, YEF	√	√	√	/
Excluded organizations					
Policy briefs	Gender and Adolescence: Global Evidence, GAGE	√	×	×	Elements not included
	Human Sciences Research Council, HSRC	√	×	×	Elements not included
	Brookings Institution	√	√	×	Elements not included
	BRAC Institute of Governance and Development, BIGD	√	√	×	Elements not included
Policy briefs	Australian National University, ANU	√	×	×	PDF file not provided
	Global HIV/AIDS Initiatives Network, GHIN	/	/	/	Updates have been stopped
Evidence-based policy briefs	Supporting the Use of Research Evidence, SURE	/	/	/	No documents are produced
Evidence briefs	The Australian Healthcare and Hospitals Association, AHHA	√	√	×	Elements not included
Briefing	Social, Technological and Environmental Pathways to Sustainability Centre, STEPS Centre	√	×	×	Elements not included
Issue briefs	United Nations Development Programme, UNDP	√	×	×	Elements not included
	Institute of Strategic Studies Islamabad, ISSI	√	√	×	Elements not included
Policy briefcase	The Abdul Latif Jameel Poverty Action Lab, J-PAL	√	√	√	One policy brief only

According to the text of the EBPs, the selection of documents for inclusion is based on identification criteria. The documents developed by the organization were qualitatively evaluated on the basis of the results of the extraction. Documents from 12 organizations were excluded from the analysis and the organizations excluded, and the reasons for exclusion are presented in Table 2. Two organizations were excluded from the analysis: GHIN, because the initiative is no longer active and its website has been discontinued, making its documents inaccessible; and SURE, because a review of its publications determined that it has not produced EBPs.There were 20 (100%) with problem components, 15 (75%) with options components, 11 (55%) with implementation considerations components. The details are presented in Table 2.

### Methodological components of EBPs

Following the application of eligibility and prioritization criteria, 10 organizations formed the core of our analysis, from which a total of 50 documents were sampled. Of the 50 included documents (5 per eligible organization), in terms of the main topic, 34 (68%) documents focused on health. In total, 10 (20%) documents addressed a social topic, such as poverty reduction and social welfare programs. A total of six (12%) covered education topics, including preventing violence and teachers’ training. Only one EBP (2%) is a living document, meaning it is continually updated.

#### Co-production for both policymakers and stakeholders (evidence demand side)

**Fig. 1:**
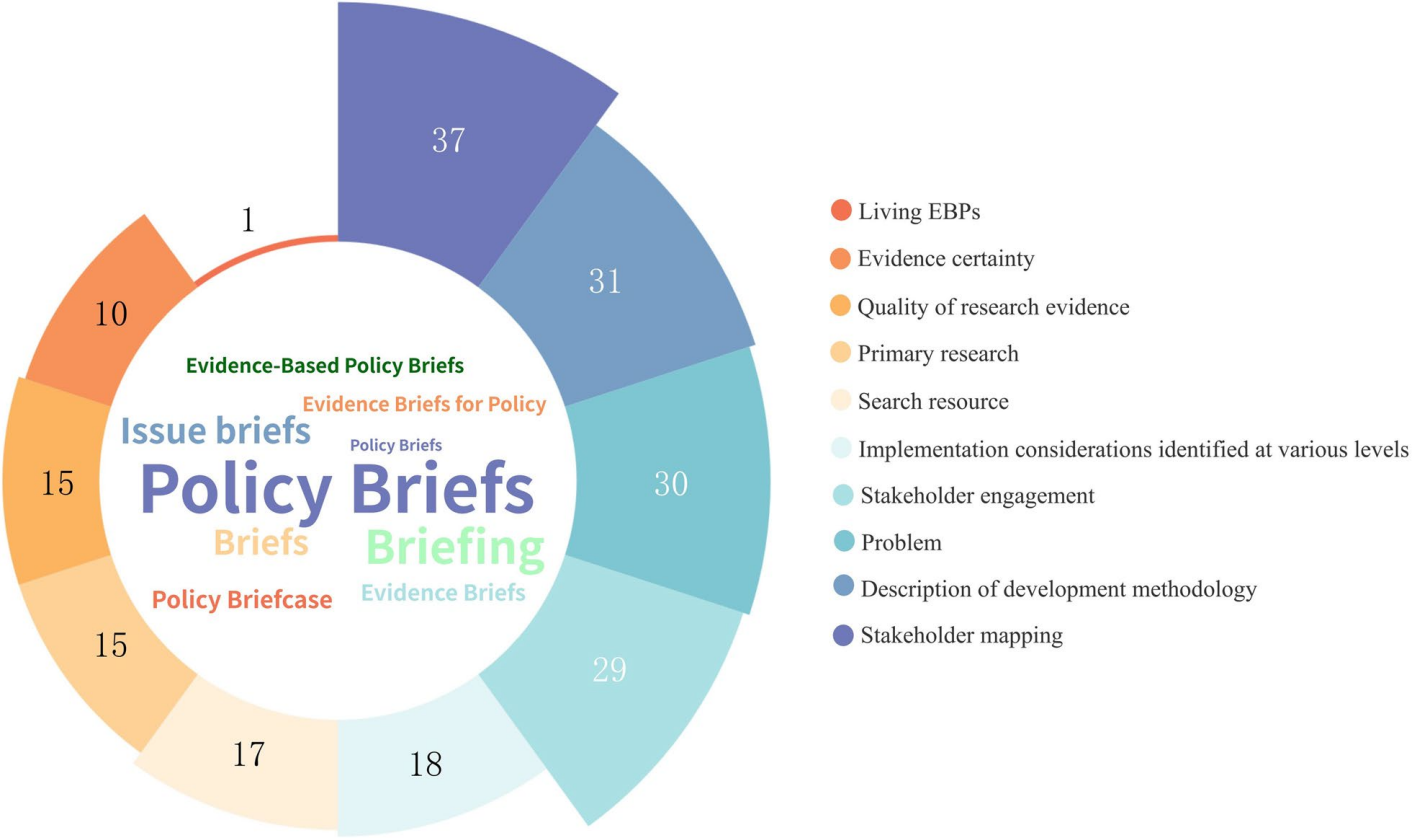
Components and methodology of EBPs

Regarding co-production, 30 (60%) documents were developed in response to demand-side requirements. A total of 29 (58%) documents described stakeholders in EBPs development through steering group, technical group and so on. In total, 18 (36%) documents identified implementation considerations at various levels. For example, these might be at the levels of patients/citizens, health workers, organizations and the system [14]. Among the literature reviewed, 27 (54%) documents developed EBPs that included both stakeholder mapping and engagement. Additionally, 10 (20%) EBPs described stakeholder mapping but did not include stakeholder engagement. Notably, there were two (4%) EBPs that addressed stakeholder engagement without detailing stakeholder mapping (Fig. 1 and Table 3).

**Table 3:** Methodological quality of EBPs with three key components^*^

Terms	Organization/institution	Main topic	Living EBPs	Co-production			Description of development methodology	Primary research	Evidence synthesis			Implementation considerations identified at various levels
				Problem^*^	Stakeholder mapping	Stakeholder engagement^*^			Search resource	Evidence certainty	Quality of research evidence	
Evidence briefs for policy	EVIPNet	Health 5	0	4	5	5	5	0	5	1	5	5
Policy briefs	K2P	Health 5	0	5	5	5	5	1	1	0	0	5
	WHO	Health 5	0	1	2	1	0	4	0	0	0	2
	OBS	Health 5	0	0	5	3	3	0	1	0	0	1
	World Bank	Social 4 Health 1	0	1	5	1	1	2	0	0	0	0
	Think7	Social 5	0	2	5	2	0	4	0	0	0	0
Evidence briefs	McMaster health forum	Health 5	1	5	5	5	5	0	5	4	5	5
	VA	Health 5	0	5	5	5	5	0	5	5	5	0
Briefs	3ie	Health 3 Social 1 Education 1	0	5	0	0	4	2	0	0	0	0
Briefing	YEF	Education 5	0	2	0	2	3	2	0	0	0	0

The numbers in the table indicate the number of samples that meet the items in the sample; the number of samples extracted from each organization/institution is five

*EVIPNet* Evidence-Informed Policy Network, *K2P* The American University of Beirut, Knowledge to Policy Center, *WHO* World Health Organization, *OBS* European Observatory on Health Systems and Policies, *VA* Department of Veterans Affairs, *3ie* International Initiative for Impact Evaluation, *YEF* The Youth Endowment Fund

#### Evidence synthesis (evidence supply side)

In total, 31 (62%) of the sampled documents described the methodology for their development, and 19 (38%) did not report the methodology. A total of 17 (34%) documents were based on evidence synthesis, and 15 (30%) documents were developed on the basis of primary research; in terms of synthesis of evidence, 17 (34%) documents described the search resource. Of the 10 (20%) documents that describe evidence certainty, 5 documents used Grades of Recommendations Assessment, Development and Evaluation (GRADE) [22], 4 documents used Agency for Healthcare Research and Quality (AHRQ) Methods Guide for Comparative Effectiveness Reviews [23] and 1 document used the Hoy et al. tool. A total of 15 (30%) documents reported the quality of research evidence. Of these, 10 documents used A Measurement Tool to Assess Systematic Reviews 2 (AMSTAR 2) or AMSTAR [24], and 5 documents used RoB 2 (Risk of Bias 2 Tool). GRADE assesses evidence quality/confidence (enhanced by meta-analyses), primarily developed for clinical recommendations. Its applicability in politics remains uncertain owing to lacking comparable frameworks and the frequent absence of meta-analyses in systematic reviews [25].

### The existing evaluation/assessment tools of EBPs

We searched Google and Google Scholar, reviewing the top 200 records of each platform using the following terms: “brief for policy”, “policy brief”, “EBP”, “evidence brief”, “issue brief”, “citizen brief”, “research brief”, “evidence-informed policy brief” and “evidence-based policy brief”. The publication date was restricted to the period from January 2009 and December 2024. To identify tools used in practice that may not be captured in the published literature, we conducted a consultation with the advisory board. Finally, four methodological studies/tools and five report studies/guidelines were identified (Table 4).

**Table 4:** Methodology quality assessment and reporting tools for EBPs

Category/publications	Organization	Year	Details
Methodology			
SUPPORT Tools for evidence-informed health Policymaking (STP) [17]	McMaster Health Forum, MHF	2009	Six questions
Evidence briefs for policy: using the integrated knowledge translation approach: guiding manual [14]	WHO Regional Office for Europe and Knowledge to Policy Center (K2P)	2020	Nine questions, each graded on a scale of 1–7
Diretriz metodológica: síntese de evidências para políticas [26]	Brasília: Ministério da Saúde	2020	21 questions, each question must be answered “yes”, “no”, “partially” or “not clear”
An evaluation of the evidence brief for policy development process in WHO EVIPNet Europe countries (Murphy A, Šubelj M, Babarczy B, et al.) [27]	EVIPNet Europe	2022	Combining semi-structured interviews and document review
Reporting			
How to write a policy brief [10]	International Development Research Centre, IDRC	2016	Five key elements of an effective structure before writing EBP
Writing and disseminating policy briefs [5]	The University of Iowa Injury Prevention Research Center, IRPC	2017	EBP writing (title, introduction, body, policy implications, recommendations and references) and disseminating
An essential guide to writing policy briefs [11]	International Centre for Policy Advocacy, ICPA	2017	Nine elements of planning and writing EBP
Development of a standard reporting guideline for evidence briefs for policy, STEP (Yu X, Wang Q, Moat KA, et al.) [13]	/	2022	Full text unpublished
Policy brief template: how to write an effective policy brief [12]	WHO Regional Office for the Eastern Mediterranean	2024	Used in the WHO Mediterranean Region only

## Discussion

This study involved a systematic survey of EBPs. The methodological components of those EBPs were described, including the three key components. Most organizations included the so-called problem component in their evidence briefs, while the inclusion of options and implementation considerations was less consistent. The variation in terminology used across organizations points to a lack of standardization in the labelling of such documents. Methodological shortcomings were identified in the EBPs from both the supply-side and demand-side perspectives, highlighting the need to validate and better implement existing tools and to complement existing guidelines.

### The methodological quality of EBPs requires enhancement

In 2009, John Lavis et al. developed SUPPORT [17] for evidence-informed health policymaking, posing six key questions at a time when there was no standard to accurately categorize the general quality of EBPs [26]. In 2020, the WHO Regional Office for Europe developed nine questions, each scored on a scale from 1 to 7, to assess the quality of EBPs, but that tool lacked questions on evidence searching and certainty [14]. In the same year, a guide for the production of EBPs was developed by the Ministry of Health in Brasília, providing a methodological quality assessment tool with 21 questions [27]. In 2022, the WHO Secretariat of EVIPNet Europe used a Rapid Appraisal tool, combining semi-structured interviews and document review to evaluate EBPs in Estonia, Hungary and Slovenia [28].

Several documents guiding EBP development have already been published [5, 10, 11]. For example, as mentioned above, the WHO issued a template to help write an effective EBP [12] and a standard reporting guideline for evidence briefs for policy (STEP) has been developed by Yu et al. [13]. However, Zhang et al. analysed 129 health-related EBPs in English and Chinese published from 2016 to 2020 and found that only about 46% described their methodology. Furthermore, only 9% reported their method of evidence synthesis, and 18% reported that they were based on database searches. While no specific tool has been validated to assess the methodological quality, some organizations have proposed checklists [29]. There is an urgent need for a tool to assess the methodological quality of EBPs.

### Promoting policymaker engagement in EBPs development

A division of labour among global and national developers of guidance and policy is essential to support evidence-informed policymaking for health systems [30]. However, researchers and policymakers have different priorities. Researchers, when developing EBPs, tend to prioritize addressing potential objections from fellow academics, who are concerned with rigour and internal validity [31]. By contrast, policymakers are more focused on issues related to generalizability, understandability and utility [31]. The co-production of these documents by stakeholders such as policymakers and community leaders suggests a trend toward more inclusive and demand-driven policy development. However, the synthesis of evidence remains a critical area for improvement. The methodological quality of EBPs is insufficient from both the demand side (policymakers and stakeholders) and the supply side (the producers of EBPs).

Policymakers using EBPs may rely on the reputation or perceived legitimacy of the producing organization and other factors to understand the degree of confidence they can place in their decision-making [32]. Critical methodological appraisal tools for EBPs can bridge the gap between scientific researchers and policymakers, enhance the use of evidence in policymaking and promote knowledge translation [33]. Such a tool would not dictate the final decision but would empower policymakers to weigh scientific evidence more accurately alongside other critical decision-making factors. However, a research gap still exists in the assessment of the methodology used in the development of EBPs, in agreement with the findings of this study [2, 28]. To date, no widely adopted methodological checklist has been established. Therefore, it is essential to develop or optimize a pragmatic, multi-purpose tool. The primary audiences for this tool would be: (1) EBPs producers (for example, research institutions, think tanks): to serve as a methodological guideline for developing briefs, ensuring they are constructed with transparency, rigour and completeness, thereby enhancing their inherent credibility. (2) Policymakers (the evidence demand side): To provide a practical evaluation framework for critically appraising the methodological quality of briefs they receive.

### Integrating living evidence into EBPs

Evidence-informed policymaking (EIP) demands that policymakers use the available best evidence to inform policy decisions systematically and transparently [34]. In that context, EBPs represent a relatively new, innovative approach to packaging research evidence for policymakers. A key step in developing an EBP is to gather all relevant local, regional and international evidence on the problem, its underlying factors and potential policy options [14].

Compared with sources of evidence that are infrequently or irregularly updated, living EBPs are a form of living evidence synthesis, which can be thought of as a report that evolves as things change and new evidence emerges over time [35]. This approach aims to ensure that policy decisions can be informed by emerging research evidence, which is particularly crucial in rapidly evolving contexts like a pandemic [36]. For example, during the coronavirus disease 2019 (COVID-19) pandemic, resources were saved by reducing the duplication of effort, and policymakers benefited from having up-to-date sources of evidence [37]. Another benefit of living EBPs is the continual, iterative interaction between EBP producers and policymakers. This interaction allows for the progressive refinement and co-production of documents and processes, ensuring they continue to meet the evolving needs of policymakers. Ongoing collaboration between policymakers and EBP producers can thus enhance policy development by fostering better awareness and understanding of different roles and perspectives [38].

### Strengths and limitations

Our study has several strengths. First, it provides an overview of the status of EBPs and the organizations that develop them, including the three key components of problem, options and implementation considerations. Second, it analyses the components included in each of these EBPs. This study offers valuable insights: an overview of EBP terminology, a methodological analysis of its components and a clearer understanding of the methodological gaps in the field.

However, this study also has its limitations. It was primarily based on publicly available EBP documents, and the authors of these documents were not contacted for verification, which could have led to biased judgments. Second, the exclusive focus on documents published in English limited the scope of the analysis, preventing a more global perspective that might have revealed significant regional variations in EBP development. Finally, and most critically, the use of a convenience sampling approach severely limits the representativeness and generalizability of the findings. The sample was restricted to readily accessible documents from a few prominent, mainstream organizations owing to the overwhelming volume of materials available.

## Authors’ conclusions

Methodological shortcomings were identified in the EBPs from both the supply-side and demand-side perspectives, highlighting the need to validate and better implement existing tools and to complement existing guidelines.

## Supplementary Information

Supplementary material 1. Search Strategy and Criteria.

Supplementary material 2. Date extract of included documents

## Data Availability

No datasets were generated or analysed during the current study.
