# Supplementary material for: Components and methodology of evidence briefs for policy: the need for evaluation tools
Source: Health Res Policy Syst. 2026 Feb 26;24:21. doi: 10.1186/s12961-026-01451-y (PMC12947396; doi:10.1186/s12961-026-01451-y)
Supplement: Supplementary file 1 — Supplementary material 1. Search Strategy and Criteria. [file 12961_2026_1451_MOESM1_ESM.docx]

**METHODS**

We used a two-stage approach to identify EBPs for this study, due to EBPs being gray literature and the specificity of their search [1]. First, organizations and institutions involved in the development of EBPs were identified, followed by an analysis of documents prepared primarily to provide policymakers with information, including both research evidence and other types of policy-relevant content.

**1.** **Literature search**

Google and Google Scholar were used as the main search engines in this study, with search terms such as “policy brief”, “evidence brief”, “issue brief”, and “brief”; the terms were continually modified to discover more production organizations. The search was conducted in English, over the time period of January 1 2019 to June 30, 2024. Then, expert consultations were conducted to verify that the organizations/institutions were valid for inclusion. Finally, five documents prepared by each organization/institution were sampled. All documents included in this study were downloaded from the website of the organization/institution, and the full texts of the included studies were collated and coded.

**2. Criteria**

According to mainstream definition, Evidence Briefs for Policy (EBPs) are prepared by synthesizing and contextualizing the best available evidence about a problem, presenting viable solutions to address it, and addressing key implementation considerations, with the involvement of content experts, policy-makers, and stakeholders. The inclusion criteria for documents in this study were as follows: 1) documents must have been prepared with the primary intention of providing information (both research evidence and other types of policy-relevant information) to policymakers and broadly aim to support policy-making, 2) must contain the three key components of problem, options, and implementation considerations, and 3) must have been developed or updated in the last five years.

**3. Screening and data extraction**

After the search process, the literature screening was completed according to whether the text of the EBPs met the three criteria. The extraction table was modified and developed with reference to the extraction entries of Adam et al. and Zhang et al^[1, 2]^, and modified through two rounds of pre-testing. Then, a pre-developed table in Microsoft Excel 2016 was used to perform the data extraction. Extracted information included, but was not limited to, the following: organizations, terms, problem, options, implementation considerations, living document, development methodology, search resource, evidence certainty, quality of research evidence, living EBPs, key informant interviews, and demand-side requests. Two researchers performed the data extraction independently, and a third researcher ruled on all disagreements between them.

**4. Data analysis**

To facilitate the analysis of the retrieved documents and to develop a deeper understanding of their characteristics and methodologies, the extracted data were integrated and statistically analyzed. Descriptive statistics were used to report and summarize the basic information of the included studies. Content analysis of the sampled documents began during the initial data extraction phase and followed an inductive thematic approach through multiple iterations to categorize the documents by content type, characteristics, labeling terminology, and methodology. The data were summarized as numbers and percentages. For the qualitative analysis of elements, a “√” was used to indicate the presence of an element, and an “×” to indicate its absence.

Based on the EBP-related development manuals and expert comments, criteria for data judgment were established as follows: 1) a “living EBP” is an EBP continually updated as a living document [3]. 2) “implementation considerations identified at various levels” indicates that the EBP identifies implementation considerations at various levels, 3) “description of development methodology” indicates that the EBP provides a methodological description of its preparation in a suitable and accessible section of the document, 4) “primary research” indicates that the development of the EBP was based on primary research or the EBP was conducted as primary research in its own right, 5) “search resource” indicates that the EBP describes search resources for the evidence synthesis process, 6) “evidence certainty” indicates that the EBP describes the certainty of the evidence for the evidence synthesis process, 7) “quality of research evidence” indicates that the EBP assesses the methodological quality of the studies included in its development process, 8) “problem” indicates that the the EBP was developed at the request of the demand side (i.e. government, society, organizations, etc.), 9) “stakeholder mapping” indicates that the EBP includes stakeholder mapping [4], and 10) “stakeholder engagement” indicates that the stakeholders engaged in development of the EBP through a steering group, a technical group, etc.

Table 1. Extraction items and description

| **Extraction items** | **Description** |
| --- | --- |
| Living EBP | The EBP continually updated as a living document [5]. |
| Implementation considerations | The EBP identifies implementation considerations at various levels. |
| Development methodology | The EBP provides a methodological description of its preparation in a suitable and accessible section of the document. |
| Primary research | The development of the EBP was based on primary research or the EBP was conducted as primary research in its own right. |
| Search resource | The EBP describes search resources for the evidence synthesis process. |
| Evidence certainty | The EBP describes the certainty of the evidence for the evidence synthesis process. |
| Quality of research evidence | The EBP assesses the methodological quality of the studies included in its development process. |
| Problem | The EBP was developed at the request of the demand side (i.e. government, society, organizations, etc.) |
| Stakeholder mapping | The EBP includes stakeholder mapping [6]. |
| Stakeholder engagement | The stakeholders engaged in development of the EBP through a steering group, a technical group, etc. |

**REFERENCES**

[1] Adam, T., Moat, KA., Ghaffar, A. et al. Towards a better understanding of the nomenclature used in information-packaging efforts to support evidence-informed policymaking in low- and middle-income countries. Implementation Sci. 2014; doi:10.1186/1748-5908-9-67.

[2] Zhang J. Reporting standard research on evidence‑based health policy brief. 2021; Available from: https://kns.cnki.net/kns8/defaultresult/index. Accessed 10 Mar 2024.

[3] Chakraborty S, Kuchenmüller T, Lavis J, et al. Implications of living evidence syntheses in health policy. Bull World Health Organ. 2024; doi:10.2471/BLT.23.290540

[4] Henwood, R. Stakeholder analysis : drawing methodological lessons from review of relevant literature. 2017; Available from: https://open.uct.ac.za/server/api/core/bitstreams/fc804753-45cb-41d9-9b6f-8e23ff509bb6/content. Accessed 6 May 2024.

[5] Shea, BJ., Grimshaw, JM., Wells, GA., et al. Development of AMSTAR: a measurement tool to assess the methodological quality of systematic reviews. 2007; doi:10.1186/1471-2288-7-10

[6] Higgins, JP., Altman, DG., Gøtzsche, PC., Jüni, P., Moher, D., Oxman, AD., Savovic, J., Schulz, KF., Weeks, L., Sterne, JA., Cochrane Bias Methods Group, & Cochrane Statistical Methods Group The Cochrane Collaboration's tool for assessing risk of bias in randomised trials. BMJ (Clinical research ed.), 2011; doi:10.1136/bmj.d5928.
